# Editorial: Highlights in psychology: cognitive bias

**DOI:** 10.3389/fpsyg.2023.1242809

**Published:** 2023-07-03

**Authors:** Sergio Da Silva, Rashmi Gupta, Dario Monzani

**Affiliations:** ^1^Department of Economics, Federal University of Santa Catarina, Florianopolis, Brazil; ^2^Cognitive and Behavioural Neuroscience Laboratory, Indian Institute of Technology Bombay, Mumbai, India; ^3^Department of Psychology, University of Palermo, Palermo, Italy

**Keywords:** cognitive bias, cognitive biases and heuristics, cognitive psychology, social psychology, psychopathology

Cognitive biases are unconscious and systematic errors in thinking that occur when people process and interpret information in their surroundings and influence their decisions and judgments (Kahneman et al., 1982). These biases can distort an individual's perception of reality, resulting in inaccurate information interpretation and rationally bounded decision-making (Kahneman, 2011). Cognitive biases may also contribute to psychotic symptoms (Garety et al., 2007). This Research Topic brings together 13 articles that address these issues.

Two papers are reviews. In the first, Berthet and de Gardelle conduct a systematic review of heuristics and biases tasks that measure individual differences and reliability in this Research Topic and provide a heuristics and biases inventory, an open-source catalog of over 40 previously published individual difference measures. This is useful because it takes time to find measures and determine their reliability. The second review on this Research Topic by Liu et al. is about negative-biased implicit memory. According to the literature, patients with current major depressive disorder have abnormal implicit memory. However, its function in current and remitted major depressive disorder patients when processing stimuli with positive, neutral, and negative emotions is unknown. The authors review and elaborate on the role of implicit memory in these patients found in meta-analyses in the Web of Science, PubMed, and EMBASE databases between 1990 and 2022. They report a general deficit in implicit memory in current patients. Furthermore, current patients' implicit memory performance to neutral stimuli is lower than controls', but recovered in remitted patients. Furthermore, both current and remitted patients have an implicit memory deficit to positive stimuli, and the implicit memory response to negative stimuli in current patients is similar to controls but worse in remitted patients. As a result, current patients' negative bias compensates for the general implicit memory deficit. With remission, the implicit memory of neutral stimuli recovers, but it remains abnormal in processing positive and negative stimuli. Therefore, abnormal implicit memory of positive and negative stimuli is relevant to the pathogenesis of depression.

There are four works in cognitive psychology. (1) The study on this Research Topic by Melnik-Leroy et al. is about the intriguing exponential bias, which is the tendency to underestimate exponential growth systematically and perceive it in linear terms. Attempts to reduce this bias in graphical representations using a logarithmic scale rather than a linear scale produce more perceptual errors. The authors show that the log scale induces more errors in graph description tasks, whereas the linear scale misleads people when predicting the future trajectory of exponential growth. However, a brief mathematical educational intervention can mitigate both scales' difficulties. (2) Polyanskaya's article on this Research Topic addresses the overconfidence bias or awareness of what one knows vs. does not know. In particular, it focuses on individuals' ability to monitor their cognitive performance and decisions. Retrospective confidence ratings are used to assess metacognitive monitoring, in which individuals are asked to report how certain they are in response or their performance in high-level cognitive or low-level perceptual tasks. Polyanskaya contends that the reliability of this measure is affected by factors such as what is being evaluated, how the confidence response is elicited, and the overall proportion of different trial types within one experimental session. It is important to consider how questions are posed and whether individuals are asked to evaluate what they know rather than what they do not know. When individuals are asked to assess positive evidence and the absence of positive evidence, retrospective confidence ratings are unreliable. (3) People frequently misestimate the probability of an event based on uncertain evidence. Various explanations for these judgment errors have been proposed. Some studies attribute the errors to underweighting the event's base rate or overweighting the evidence for the individual event. The paper on this Research Topic by Branch and Hegdé examines the contributions of potential explanatory variables to probability judgments under four different problem scenarios. They discovered that the explanatory variables accounted for ~30–45% of the overall variance of responses, depending on the problem scenario. No single factor can explain more than 53% of the explainable variance, let alone all of it. They conclude that attributing probabilistic judgment errors to any cause, including base rate neglect, is statistically untenable. A more nuanced explanation is that actual biases result from a weighted combination of multiple contributing factors, the exact mix of which depends on the problem scenario. (4) In this Research Topic, Suomala and Kauttonen define computational meaningfulness as the ability of humans to make a situation understandable to respond optimally. Computational meaningfulness takes into account multidimensional and changing settings. As a result, computational meaningfulness should moderate biases. Using the confirmation bias and the framing effect as examples, the authors argue that computational meaningfulness implies that these biases are necessary for optimal decision-making and can thus be deemed rational from this standpoint. The authors propose using naturalistic stimuli, such as vignettes, to build more realistic decision-making study environments and evaluate the resulting data with machine learning to improve behavior modeling.

Four articles on this Research Topic are concerned with social psychology. (1) Meng and Feng's paper on this Research Topic investigates the optimistic bias in young online taxi users who must choose between convenience and privacy in digital travel platforms. Using a model of protective motivation theory, the authors investigate the moderating effect of user knowledge of privacy settings on the relationship between privacy concerns and protective behavior. They discovered that increased privacy-protective behavior is associated with privacy concerns and positively related to perceived threats, self-efficacy, and response efficacy. As a result, there is an optimistic bias in privacy management. Previous research on the impact of intuitive-deliberative cognitive style and risk style on risky choice framing has yielded conflicting results. (2) Wyszynski and Diederich consider a psychophysical data collection approach in this Research Topic and discover that framing effects strength, cognitive style, and risk style are related. They conduct two studies, one of which counts the number of frame-inconsistent choices, and the other compares the proportions of risky choices on gain-loss frames. They vary the number of people affected, the chances of surviving or dying from an unusual disease, the type of disease, and the response deadlines. They find that risk style moderates the framing effect on the proportion of risky choices, while cognitive style in one of the studies moderates the framing effect. However, they find no link between the number of frame-inconsistent choices and cognitive or risk styles. (3) The article on this Research Topic by Korteling et al. contends that perceptions of sustainability problems, such as climate change, do not lead to sustainable choices due to cognitive biases. They list social-psychological dimensions common to most sustainability issues, such as experiential ambiguity, long-term consequences, complexity and uncertainty, threats to the status quo and social status, social dilemmas, and group pressure. They match corresponding cognitive biases using a neuro-evolutionary perspective for each characteristic. These are evolved biases that influence our preferences and behavior. They then propose interventions such as incentives and nudges to help people make more sustainable choices. (4) Naive realism is the tendency to believe that our subjective experience of reality is objective and that others should naturally perceive the world as we do. The bias that others share one's knowledge entails the curse of knowledge, which is the inability to fathom the reasoning of those who do not share one's knowledge. In this Research Topic, Beattie and Beattie apply the curse of knowledge to political cognition. They argue that overestimating the knowledge of political opponents is associated with more negative evaluations. As a result, opponents take on the character of someone who understands why an opposing viewpoint is correct but continues to oppose it. This results in political polarization. Fortunately, in a debiasing experiment, the authors discover that participants who receive an epistemological treatment evaluate those with whom they disagree more favorably.

The final three papers focus on psychopathology. (1) The paper on this Research Topic by Blauth and Iffland addresses the attentional biases associated with maltreatment and victimization experiences in childhood and adolescence. Using an online version of the facial dot-probe task, they investigate attentional processes for emotional facial expressions (anger, disgust, happiness, and sadness) in an adult sample. They discovered that attentional biases in child maltreatment are associated with angry facial expressions, which can be interpreted as threat-related biases. In contrast, biases in the context of peer victimization are related to sad facial expressions, indicating a mood-congruent bias. (2) Overcoming persistent negative thoughts is one obstacle in encouraging depressed individuals to seek assistance. In this Research Topic, Keeler et al. conducted two randomized pre-post trials to determine whether a novel online intervention employing mental contrasting and implementation intentions could increase actual help-seeking or the intent to seek help for depression. The study takes into account self-reports from individuals in the United States. These trials show the viability and preliminary success of such an intervention to encourage help-seeking, which may be helpful to clinicians. (3) According to the cognitive model of psychosis, psychotic symptoms may originate from biased information processing. The study in this Research Topic by Sanchez-gistau et al. focuses on the differences in selected cognitive biases (intentionalizing, catastrophizing, dichotomous thinking, jumping to conclusions, and emotional reasoning) between individuals experiencing first-episode psychosis (FEP) with and without comorbid attention deficit and hyperactivity disorder (ADHD). The researchers use the Cognitive Biases Questionnaire for Psychosis to assess the severity and types of cognitive biases in FEP-ADHD+, FEP-ADHD-, and healthy controls (HCs). According to the findings, FEP-ADHD+ participants have considerably greater cognitive biases than FEP-ADHD- individuals and HCs. In particular, the FEP-ADHD+ group is more strongly related to intentionalizing and emotional reasoning biases. Cognitive biases are associated with positive psychotic symptoms in both groups but only with depressive symptoms in the FEP-ADHD- group and impaired functioning in the FEP-ADHD+ group. These findings imply that FEP-ADHD+ individuals may require focused metacognitive interventions. The study emphasizes the necessity of treating FEP with ADHD and recommends more research to develop individualized pharmacological and psychological interventions for specific FEP subpopulations.

In conclusion, the publications on this Research Topic demonstrate the rich diversity of theoretical and empirical findings across a broad spectrum of contemporary cognitive bias research. Cognitive biases are significant because they can influence how individuals perceive and interpret information, which may or may not lead to judgment and decision-making errors. Researchers must comprehend cognitive biases to develop interventions designed to improve decision-making and mental health. We are grateful to all those who took the time to provide insightful input. We hope that their research will inspire future investigation.

## Author contributions

All authors listed have made a substantial, direct, and intellectual contribution to the work and approved it for publication.

## References

[B1] Garety P.A. Bebbington P. Fowler D. Freeman D. Kuipers E. (2007). Implications for neurobiological research of cognitive models of psychosis: a theoretical paper. Psychol. Med. 37, 1377–1391. 10.1017/S003329170700013X 17335638

[B2] Kahneman (2011). Thinking, Fast and Slow. New York, NY: Farrar, Straus and Giroux.

[B3] Kahneman D. Slovic P. Tversky A. (eds.). (1982). Judgment Under Uncertainty: Heuristics and Biases. New York, NY: Cambridge University Press. 10.1126/science.185.4157.1124 17835457

